# Identifying competencies required for medication prescribing for general practice residents: a nominal group technique study

**DOI:** 10.1186/1471-2296-15-139

**Published:** 2014-08-01

**Authors:** Jean-Pascal Fournier, Brigitte Escourrou, Julie Dupouy, Michel Bismuth, Jordan Birebent, Rachel Simmons, Jean-Christophe Poutrain, Stéphane Oustric

**Affiliations:** 1 Département Universitaire de Médecine Générale, Faculté de Médecine, Université Paul Sabatier, Toulouse, France; 2 UMR 1027, Inserm, Toulouse, France; 3 Department of Family Medicine, McGill University, Montreal, Quebec, Canada

**Keywords:** Medical education, Consensus, Curriculum, General practice, Drug prescriptions

## Abstract

**Background:**

Teaching of medication prescribing is a specific challenge in general practice curriculum. The aim of this study was to identify and rank the competencies required for prescribing medication for general practice residents in France.

**Methods:**

Qualitative consensus study using the nominal group technique. We invited different stakeholders of the general practice curriculum and medication use in primary care to a series of meetings. The nominal group technique allowed for the quick development of a list of consensual and ranked answers to the following question: “At the end of their general practice curriculum, in terms of medication prescribing, what should residents be able to do?”.

**Results:**

Four meetings were held that involved a total of 31 participants, enabling the creation of a final list of 29 ranked items, grouped in 4 domains. The four domains identified were ‘pharmacology’, ‘regulatory standards’, ‘therapeutics’, and ‘communication (both with patients and healthcare professionals)’. Overall, the five items the most highly valued across the four meetings were: ‘write a legible and understandable prescription’, ‘identify specific populations’, ‘prescribe the doses and durations following the indication’, ‘explain a lack of medication prescription to the patient’, ‘decline inappropriate medication request’. The ‘communication skills’ domain was the domain with the highest number of items (10 items), and with the most highly-valued items.

**Conclusion:**

The study results suggest a need for developing general practice residents’ communication skills regarding medication prescribing.

## Background

Medication prescribing is one of the most common activities during general practice visits. In Europe, rates of medication prescription per general practice visit range from 43% to 90%, France being the country with the highest prescription rate per general practice visit [1]. Prescribing is a complex process and 4.9% of all prescription items have been found to include errors in prescribing or monitoring in general practice, with 0.2% of prescriptions involving severe errors [2]. Moreover, every step of medication use in primary care has been associated with sub-optimal processes resulting in only 4% to 21% of patients achieving maximum benefit from their medication [3].

Prescribing medication is a complex task requiring the understanding of basic principles of clinical pharmacology and therapeutics, the knowledge of medicines, the application of different skills (diagnostic, communication), the appreciation of risk and uncertainty, and, ideally, ample experience in clinical practice [4]. In these regards, teaching of medication prescribing is a specific challenge in post-graduate medical education. Evidence indicates this may be insufficient, as junior doctors tend to make more errors than their older colleagues [5-7]. Moreover, newly graduated physicians declare they are unprepared to safely prescribe medication at the beginning of their residency year [8,9]. Newly graduated physicians have identified prescribing as their ‘weakest area of practice’ during their first post-graduation year [9].

The general practice curriculum in France is based on a competency framework entitled ‘référentiel métiers et compétences des médecins généralistes’, that has been developed by the National College of Teaching General Practitioners [10]. This framework was designed after extended observations of visits in general practice by a multidisciplinary team. It describes, through typical medical encounters, the competencies required to practice as a general practitioner. Medication prescribing in general practice and prescribing-related competencies are however not specifically addressed in this framework. Additionally, the perspectives of other health professionals could provide valuable insights on medication prescribing teaching for general practice residents. It has already been shown that primary care providers (other than general practitioners) could help identify causes of prescribing errors in general practice [11], or initiatives for reducing general practitioners’ prescribing workload in rural areas [12].

Outside France, other general practice curricula have addressed the issue of medication prescribing-competencies. In the UK curriculum produced by the Royal College of General Practitioners (RCGP) [13], the ‘Patient Safety and Quality of Care’ section underlines that “prescribing and monitoring of medication needs to be understood, developed and explored to ensure high-quality, safe care”. Item 1.7 of the ‘Enhancing Professional Knowledge’ section states that medication prescribing should adhere to the General Medical Council’s principles of good medical prescribing, without providing further details. To our knowledge, the most comprehensive curriculum on the topic is the one from the Royal Australian College of General Practitioners (RACGP) [14]. The ‘Quality use of medicines’ section provides 41 training outcomes distributed in the five domains of general practice defined by the RACGP.

Also, two recent guidelines provide more detailed competency frameworks for medication prescribing. In the UK, the National Prescribing Centre has developed a framework for all prescribers (72 items classified in 9 domains), resulting from the consolidation of three pre-existing frameworks and an updated literature review [15]. In Australia, NPS MedecineWise proposes a framework of competencies required to prescribe medicines [16] classified in 5 domains, based on the prescribing guide formerly developed by the Word Health Organization [17]. It is unclear, however, how these guidelines apply to specific general practice issues or the teaching of general practice. Indeed, the Australian guideline states that the proposed framework is “not a curriculum”, and that it does “not extend to the specialized competencies required by some groups of prescribers”.

It is highly uncertain how these curricula and frameworks could be adapted for the French general practice curriculum and health care system. Additionally, they do not provide any prioritization amongst medication prescribing competencies. Thus, we conducted a qualitative study aiming to identify and prioritize the competencies required for medication prescribing for general practice residents in France.

## Methods

### Study design

This study used the nominal group technique. This technique is a qualitative method used to achieve consensus [18,19]. The nominal group technique allows for the quick development of a list of consensual and ranked answers to a precise question, following a brief meeting (45 to 120 minutes) of 6 to 12 participants. This method has been used extensively for a wide range of general practice related purposes, including exploration of emergent concepts or identification of educational needs [20-23].

The nominal group technique offers certain advantages over other consensus methods that were valuable in the context of the present study. The nominal group technique is an exploratory tool used to generate ideas when the evidence base is limited, as in the case of the present study. Also, the nominal group technique output is ranked, allowing a prioritization that was a part of the research objective. Lastly, it has a structured design, ensuring that no one participant in the group dominates the discussion and that the group facilitator has no influence on the process.

### Participant sampling

The efficacy of the nominal group technique relies on the representative sampling of participants relevant to the explored issue. We invited different stakeholders of the general practice curriculum and medication use in primary care: general practitioners (including teaching general practitioners), general practice residents, clinical pharmacologists, community pharmacists and medical officers of the Health Insurance System. At least one representative from each field was present at each meeting.

### Nominal group meetings

From October 2012 to March 2013, a series of meetings were held in an unaffiliated location within Toulouse University. The meetings were standardized and followed four steps.

First, a facilitator briefly provided an overview of the study. This presentation described the concerns of residents regarding the safety of their medication prescribing, and the necessity of identifying priorities in medication-prescribing competencies to enhance teaching. The facilitator then explained the different steps of the nominal group technique. The presentation did not have any content that could influence participants.

The following question was then posed: « At the end of their general practice curriculum, in terms of medication prescribing, what should residents be able to do? ». This question had been previously tested in a pilot sample of participants.

The participants were asked to silently write down their answers to this question, without conferring with each other. Each item was subsequently recorded in a round-robin fashion and displayed to the group using a projector. The participants then discussed the list of items. A trained facilitator made sure every participant expressed their ideas and that the rules of nominal group technique were respected. The items were clarified and similar items were merged if necessary. Items were grouped into domains, and an agreed upon list of medication-prescribing competencies was created.

Participants were then asked to anonymously assign a score indicating the importance they gave to each item (out of 10). Items were ranked according to the sum of participants’ scores. Meetings were repeated with different participants until saturation, and the lists of domains and items were compiled into a final list. The final list was sent electronically to all previous participants. They were asked to follow the same procedure as in the original meetings (assigning a score to each item on the final list), allowing for a final ranking of the items. The items with the lowest final rank were the most highly-valued.

### Ethics

Ethics approval for this study was obtained from the ‘*Commission Ethique du Département de Médecine Générale de Midi-Pyrénées’* ethics committee. Participation in the study was entirely voluntary; there was no monetary reward for participation. Informed consent was obtained from all participants. The data remains anonymized and confidential.

## Results

Four meetings were held, involving a total of 31 participants. Table 1 shows the main characteristics of the participants. Each profession was systematically represented at every meeting. Every meeting, excepting the first, lasted less than two hours (range: 1 h45 to 2 h10). At the end of the first, second, third and fourth meeting, lists were generated that contained 40, 41, 44 and 43 items respectively. The compilation and the final ranking of these items resulted in a final list of 29 items, grouped in 4 domains, and ranked by importance. The four domains identified were ‘pharmacology’, ‘regulatory standards’, ‘therapeutics’, and ‘communication’ (both with patients and healthcare professionals). Table 2 show the complete list, with the rank attributed at the end of each meeting and the final rank for each item. A condensed list (in English and in French) is available in Additional file 1.

**Table 1:** Characteristics of participants of the 4 meetings

**Characteristics**		**Number (%) (total = 31)**
Profession		
	General practitioner	8 (25.8%)
	General practice resident	8 (25.8%)
	Community pharmacist	5 (16.1%)
	Pharmacologist	5 (16.1%)
	Medical Officer of the Health Insurance System	5 (16.1%)
Years since completing training (for all participants but general practice resident, n = 23)		
	0–10	3 (13.0%)
	10-20	4 (16.6%)
	>20	16 (69.6%)
Year in general practice curriculum (for general practice resident, n = 8)		
	1st year	3 (37.5%)
	2nd year	1 (12.5%)
	3rd year	4 (50.0%)
Gender		
	Female	18 (58.1%)
Age		
	<30	7 (22.6%)
	30-40	5 (16.1%)
	40-50	6 (19.4%)
	>50	13 (41.9%)

**Table 2:** Medication prescribing-related competencies (grouped by domains) and ranks*

**Domains - items**	**Meeting 1**	**Meeting 2**	**Meeting 3**	**Meeting 4**	Final rank*
	**10 participants**	**7 participants**	**7 participants**	**7 participants**	
***Pharmacology***					
Prescribe the doses and durations following the indication	-	5	8	2	**3**
Know where to find validated information for medication prescription	12	13	10	5	**6**
Identify adverse drug reactions	-	8	11	10	**10**
Be critical with new medications	3	4	-	17	**12**
Identify potential drug interactions	5	3	8	12	**12**
Prescribe in international nonproprietary names	6	22	-	6	**24**
Prescribe in compliance to marketing authorizations	19	19	-	6	**25**
***Regulatory standards***					
Write a legible and understandable prescription for the patient and the one who administers the medication	-	1	1	2	**1**
Use two-part prescription forms for patients with chronic condition falling under *Affections de Longue Durée*†	18	5	8	3	**10**
Abide by the terms of use for specific prescriptions: secured forms, special-status medication‡ , restricted prescription, unreimbursed prescription	21	5	6	3	**23**
Include mandatory information of the prescription: identification of prescriber, date, patient’s name, age, weight (for children)	3	17	1	-	**26**
Know the costs associated with medication prescription: reimbursement rate and patient’s contribution	23	20	21	-	**29**
***Therapeutics***					
Identify specific populations (paediatric, pregnant, breastfeeding, elderly, renal impaired)	8	-	1	1	**2**
Regularly re-evaluate chronic medication prescriptions	6	4	19	13	**9**
Prescribe non-pharmacological treatment (lifestyle habits, dietary changes, physical activity, reassuring advices) over medication	1	-	18	19	**15**
Deprescribe	11	10	19	-	**18**
Abstain from systematic medication prescription	1	2	17	-	**19**
Unifies prescription from different sources	7	10	7	-	**20**
Use medication prescription software	22	21	13	20	**28**
***Communication***					
***With patient***					
Explain a lack of medication prescription to the patient	8	13	-	18	**3**
Decline inappropriate medication request for prescription medication	16	-	20	16	**5**
Explain to the patient his/her medication prescription	8	12	5	8	**7**
Assess patient’s adherence	13	13	16	-	**12**
Identify barriers to medication use	-	13	4	-	**16**
Assess self-medication	16	8	14	15	**21**
Explain potential adverse drug reactions to the patient	-	18	14	9	**22**
***With health professionals***					
Being critical about the information supplied by the pharmaceutical industry	15	-	22	11	**8**
Prescribe in collaboration with other health professionals: physicians, pharmacists, pharmacovigilance centres, Health Insurance System representatives, nurses, midwives	13	23	23	13	**16**
Report adverse drug events to Pharmacovigilance Centre	20	-	12	20	**27**

*Items with the lowest ranks were the most highly valued.

† Affection de Longue Durée: in France, a list of 30 serious chronic conditions (=Affection de Longue Durée) allows 100% reimbursement for health care related to these conditions. Specific two-part prescription forms are needed to identify which medications are related to the ALD (upper part of the form) and should be 100% reimbursed to the patient.

‡ Special-status medications include highly expensive medications, reimbursed only in very restrictive indications, in accordance with a fixed ‘special status medication list’. A specific form is needed for their prescription.

- Item not mentioned within that specific group.

Overall, the five items most valued were: ‘write a legible and understandable prescription’, ‘identify specific populations’, ‘prescribe the doses and durations following the indication’, ‘explain a lack of medication prescription to the patient’, ‘decline medication inappropriate requests’. They were not, however, identified in every meeting.

‘Communication skills’ was the domain with the highest number of items (10 items), and with the most highly-valued items (4 of the 8 most highly-valued items: ‘explain a lack of medication prescription’, ‘decline medication inappropriate request’, ‘explain to the patient his/her medication prescription’, ‘being critical about the information supplied by the pharmaceutical industry’). In contrast, the ‘regulatory aspects’ domain was the domain with the most least-valued items (3 of the 5 least highly-valued items: ‘abide by the terms of use for specific prescriptions’, ‘include mandatory information of the prescription’, ‘know the costs associated with medication prescription’).

## Discussion

Our study identified and prioritized medication prescribing competencies that are necessary for general practice residents in France. We demonstrated that a list of 29 items, grouped in 4 domains, could provide a prioritization of prescribing competencies that need to be taught in post-graduate education.

Our study provides valuable insights into the complexity of teaching medication prescribing to residents in the general practice curriculum. The importance of the ‘communication’ domain underlines the necessity of conceiving the teaching of medication prescribing in a patient-centred manner, with emphasis on patient education. Several other studies have underlined how communication about medication is poor and varies widely during medical encounters in general practice [24-26]. These findings and our results support the need for a specific evaluation of residents’ communication skills (centred on medication prescribing) to identify potential for any educational interventions.

Our results specifically stress the importance of communication in the context of the absence of a prescription (‘explains an absence of medication prescription to the patient’, ‘decline inappropriate medication request’). Junior doctors may face the ‘pressure to prescribe’ [27] whether coming from patients [28], family [29], or nursing staff [30], with insufficient training to handle these situations. Paternetti *et al.* have identified strategies used by general practitioners during medical encounters to deny patients inappropriate requests [31]. Communicating these strategies with residents and helping them applying these strategies could be beneficial to address these particular competencies.

The participants also identified new challenges in teaching of medication prescribing in primary care, especially the necessity to ‘prescribe in collaboration with other health professionals’ and unify ‘prescription from different sources’ to ensure the continuity of medication information in primary care [32]. Prescribing in collaboration is also a challenge in the current context of reorganization of primary care that evolves towards grouping different professionals in the same health care centres [33-35].

A recent systematic review has identified 47 studies assessing education-based interventions to aid improvement in prescribing competencies [36]. Most of the interventions were targeted at medical students, residents or general practitioners. Some of these interventions addressed items on our list, but focused mainly on pharmacology, therapeutics or regulatory domains; few focused on communication skills. Also, the heterogeneity of contexts, interventions and outcomes (medication prescribing competences or performances) makes it uncertain what the effects of integration of such interventions in the general practice curriculum would be, and if supplementary evaluation to properly ascertain their merit would be required.

Interestingly, the UK RCGP curriculum has been updated during the study period, introducing emphases on medication prescribing-related competences [13]. The following three items have been added to the Patient Safety and Quality of Care’ section: ‘demonstrate an understanding of the principles of medicines management’, ‘describe how to report adverse drug reactions and clinically significant errors through the appropriate national reporting systems’ and ‘provide patients with information on the risks and benefits of treatments to allow them to make informed decisions’. The last two items have been identified by the participants of our study, although expressed differently. Also, our list shares elements that are similar to those of the curriculum from the RACGP [14]: two of the five domains are identical (communication skills and regulatory/legal aspects), and the majority of the items are common, although expressed differently. Some discrepancies include items referring to specificities of the Australian and French health care systems. The RACGP curriculum underlines some points that have not been identified by the participants, such as the importance to take into account health literacy, culture and language influences when prescribing medication, or a focus on prescribing of antimicrobial agents. Also, some points cited by the participants are not found in RACGP curriculum (‘prescribe in international nonproprietary names’, ‘deprescribe’, or ‘re-evaluate chronic medications).

Our list contains fewer details (29 items) than the two UK and Australian prescribing frameworks [15,16]. Some items of our list are singular, since they do not appear elsewhere in these two frameworks. Certain aspects can be attributed to the specificity of the French context, such as the use of the ‘two-part prescription forms’ [two-part prescription forms are needed to identify medications related to the serious conditions (found in the upper part of the form) that allow full reimbursement to the patient] and reporting Adverse Drug Reactions (ADR). In France, it is mandatory to report ‘unexpected’ ADR (unlabelled) or ‘serious’ ADR (lethal, life-threatening, requiring hospitalization or hospitalization prolongation, or causing persistent or significant disability/incapacity). This item, though ranked as less important, appears necessary considering the decrease in participation of general practitioners for reporting ADRs [37]. The other items on our list were all identified in the two guidelines, though expressed differently (e.g. ‘prescribe in international nonproprietary names’ in our list corresponds to ‘prescribes generically where appropriate, practical and safe for the patient’ in the UK guideline, and ‘uses the active ingredient name of the medicine’ in the Australian guideline). On the other hand, some domains or items are absent on our list: in particular, shared-decision making is not clearly identified, and some steps of this process are not listed (assessing patient’s preferences, negotiating, ensuring a common understanding). Another aspect of the UK and Australian guidelines not identified on our list is the engagement of the prescriber to continual quality improvements. Lastly, whereas the UK and Australian guidelines provide competency frameworks (allowing assessment of progression from knowledge to performance in practice [38]), our final list is a combination of knowledge, competencies and actions, with no performance items. A contextualization of the items is now needed to allow the use of our preliminary list for assessment in medical education.

Some of the items on our list are common to a UK safety checklist for early specialty training in general practice, validated by Bowie *et al.* through mixed methods [39]. In this checklist, the ‘prescribing safely’ section emphasized the necessity of basic knowledge on high-risk medication, awareness of Health Board/Formulary Prescribing Guidance (corresponding to the item ‘know where to find validated information for medication prescription’), or monitoring of side-effects. Interestingly, our study did not identify any items related to ‘risks associated with signing repeat and special requests without consulting records’ [39].

The main strength of our study relies on the choice of the technique used. The structure of the nominal group technique allows participants to provide their ideas without constraint. This aspect was of high importance in the context of our study, to avoid a potential "pressure to conform" from group members towards higher status participants (e.g. residents and teaching general practitioners). Additionally, the way we chose the participants enabled the creation of a panel largely representative for the topic. We were able to incorporate the views and ideas of the two professions with the greatest daily experience in the field of medication prescribing (general practitioners and community pharmacists), as well as those of experts with a more theoretical academic or regulatory background (pharmacologists and officers of the Health Insurance System), and those of the principal party involved in the general practice curriculum (residents). To place the focus solely on academics or general practitioners would have restricted the conclusions of the study. Also, we were able to lead repeated meetings, allowing the enrolment of a large number of participants. The use of several meetings enabled the comparison of four preliminary lists and their compilation into a single final list, thus strengthening the consensus surrounding the study results. Finally, we produced a ranked list, enabling prioritization for curriculum building.

The nominal group technique, by definition, is used for consensus elaboration. Accordingly, the use of this technique implied that some potentially innovative answers were eliminated, when they were not suggested in the majority of the meetings. Secondly, some will consider our selection of participants to not be fully representative of the topic we have explored. Indeed, we did not invite any patients, nurses or midwives, whose opinions could be considered as highly relevant to our topic. Initially, we considered the possibility of involving patients in our study, since the expert patient technique (or ‘consumer approach’) has been suggested as an aid to curriculum [40]. This choice could be relevant when exploring issues of specific diseases [41]. However we considered the topic of our study to be too broad for relevant patient input. Regarding nurses or midwives, the possibility had also been discussed initially but rejected due to the complex organization of the meetings. Further studies would be needed to investigate the interaction of paramedical professionals and patients on the topic of medication prescribing in general practice.

## Conclusion

This preliminary study has identified and ranked medication prescribing competencies that should be focused on in the general practice curriculum. Our results corroborate with elements of medication-prescribing competency frameworks, as well as general practice curricula. Our results further suggest a need for developing general practice residents’ communication skills regarding medication prescribing, especially in the context of an absence of a prescription.

## Competing interests

The authors declare no competing interest.

## Authors’ contributions

JPF is the primary author who was responsible for study design, data collection, data analysis and manuscript preparation. BE designed the study, facilitated the meetings and analysed the data. SO is the primary author’s direct supervisor. JD, MB, JB, RS, JCP are contributing authors. All authors contributed significantly in the conception of the design and the interpretation of the data. All authors contributed to the manuscript and assessed it critically for scientific soundness and intellectual content. All authors revised and approved the final version for publication.

## Pre-publication history

The pre-publication history for this paper can be accessed here:

http://www.biomedcentral.com/1471-2296/15/139/prepub

## Supplementary Material

Additional file 1 Additional Tables Medication prescribing-related competencies (grouped by domains) and ranks, without the results within groups, in English and in French. Click here for file
