# Supplementary material for: Identifying competencies required for medication prescribing for general practice residents: a nominal group technique study
Source: BMC Fam Pract. 2014 Aug 1;15:139. doi: 10.1186/1471-2296-15-139 (PMC4129426; doi:10.1186/1471-2296-15-139)
Supplement: Additional file 1 — Additional Tables Medication prescribing-related competencies (grouped by domains) and ranks, without the results within groups, in English and in French. [file 1471-2296-15-139-S1.docx]

Additional Table 1: Medication prescribing-related competencies (grouped by domains) and ranks

| **Domains - Items** | **Global rank** |
| --- | --- |
| ***Pharmacology*** |  |
| Prescribe the doses and durations following the indication | **3** |
| Know where to find validated information for medication prescription | **6** |
| Identify Adverse Drug Reactions | **10** |
| Be critical with new medications | **12** |
| Identify potential drug interactions | **12** |
| Prescribe in international nonproprietary names | **24** |
| Prescribe in compliance to marketing authorizations | **25** |
| ***Regulatory standards*** |  |
| Write a legible and understandable prescription for the patient the one who administers the medication | **1** |
| Use two-part prescription forms for patients with chronic condition falling under *Affections de Longue Durée** | **10** |
| Abide by the terms of use for specific prescriptions: secured forms, special-status medication†, restricted prescription, unreimbursed prescription | **23** |
| Include mandatory information of the prescription: identification of prescriber, date, patient’s name, age, weight (for children) | **26** |
| Know the costs associated with medication prescription: reimbursement rate and patient’s contribution | **29** |

* Affection de Longue Durée: in France, a list of 30 serious chronic conditions (=Affection de Longue Durée) allows 100% reimbursement for health care related to these conditions. Specific two-part prescription forms are needed to identify which medications are related to the ALD (upper part of the form) and should be 100% reimbursed to the patient.

† special-status medications include highly expensive medications, reimbursed only in very restrictive indications, in accordance with a fixed ‘special status medication list’. A specific form is needed for their prescription.

Additional Table 1: Medication prescribing-related competencies (grouped by domains) and ranks (continued)

| **Domains - Items** | **Global rank** |
| --- | --- |
| ***Therapeutics*** |  |
| Identify specific populations (paediatric, pregnant, breastfeeding, elderly, renal impaired) | **3** |
| Regularly re-evaluate chronic medication prescriptions | **6** |
| Prescribe non-pharmacological treatment (lifestyle habits, dietary changes, physical activity, reassuring advices) over medication | **10** |
| Deprescribe | **12** |
| Abstain from systematic medication prescription | **12** |
| Unifies prescription from different sources | **24** |
| Use medication prescription software | **25** |
| ***Communication*** |  |
| ***With patient*** |  |
| Explain a lack of medication prescription to the patient | **3** |
| Decline inappropriate requests for prescription medication | **5** |
| Explain to the patient his/her medication prescription | **7** |
| Assess patient’s adherence | **12** |
| Identify barriers to medication use | **16** |
| Assess self-medication | **21** |
| Explain potential adverse drug reactions to the patient | **22** |
| ***With health professionals*** |  |
| Being critical about the information supplied by the pharmaceutical industry | **8** |
| Prescribe in collaboration with other health professionals: physicians, pharmacists, pharmacovigilance centres, Health Insurance System representatives, nurses, midwives | **16** |
| Report adverse drug events to Pharmacovigilance Centre | **27** |

Additional Table 2: Medication prescribing-related competencies (grouped by domains) and ranks (in French)

| **Domaines - Items** | **Global rank** |
| --- | --- |
| ***Pharmacologie*** |  |
| Prescrire aux posologies et durées adaptées à l'indication | **3** |
| Savoir où trouver les sources d'information validées pour la prescription médicamenteuse | **6** |
| Identifier les effets indésirables médicamenteux | **10** |
| Être critique avec les nouveautés médicamenteuses | **12** |
| Identifier les interactions médicamenteuses potentielles | **12** |
| Prescrire en dénomination commune internationale | **24** |
| Prescrire dans le cadre de l'autorisation de mise sur le marché | **25** |
| ***Regulatory standards*** |  |
| Rédiger une ordonnance lisible et compréhensible pour le patient et celui qui délivre | **1** |
| Utiliser les ordonnances bizones dans le cadre d'une affection de longue durée | **10** |
| Respecter les modalités de prescriptions particulières: ordonnance sécurisée, médicaments d'exception, prescription restreinte, non remboursée | **23** |
| Respecter les champs réglementaires de l'ordonnance: identification prescripteur: date, nom patient, âge, poids (si enfant) | **26** |
| Connaitre les coûts de ses prescriptions médicamenteuses: taux de remboursement, reste à charge du patient | **29** |

Additional Table 2: Medication prescribing-related competencies (grouped by domains) and ranks (in French, continued)

| **Domaines - Items** | **Global rank** |
| --- | --- |
| ***Thérapeutique*** |  |
| Identifier les populations spécifiques (pédiatrie, femme enceinte, allaitante, sujet âgé, insuffisant rénal) | **2** |
| Réévaluer régulièrement les prescriptions médicamenteuses chroniques | **9** |
| Prescrire du non-médicamenteux (règles hygiéno-diététiques, activité physique, conseils réassurance) plutôt que des médicaments | **15** |
| Déprescrire | **18** |
| Ne pas prescrire de médicament systématiquement | **19** |
| Centraliser les ordonnances du patient | **20** |
| Utiliser les logiciels d'aide à la prescription | **28** |
| ***Communication*** |  |
| ***Avec le patient*** |  |
| Expliquer au patient une absence de prescription médicamenteuse | **3** |
| Ne pas céder à une demande de prescription médicamenteuse inappropriée | **5** |
| Expliquer au patient son ordonnance médicamenteuse | **7** |
| Évaluer l'observance | **12** |
| Identifier les freins à la prise médicamenteuse | **16** |
| Évaluer l'automédication | **21** |
| Expliquer au patient les effets indésirables potentiels | **22** |
| ***Avec les professionnels de santé*** |  |
| Être critique devant l'information émanant des laboratoires | **8** |
| Prescrire en collaboration avec les autres professionnels de santé: médecin, pharmacien d’officine, centre régional de pharmacovigilance, service médical de l’assurance maladie, infirmière, sage-femme | **16** |
| Déclarer un événement indésirable au système de pharmacovigilance | **27** |
